# Sinus floor elevation with platelet-rich fibrin alone: A Clinical retrospective study of 1-7 years

**DOI:** 10.4317/jced.55113

**Published:** 2018-10-01

**Authors:** Naofumi Aoki, Michinori Maeda, Masashi Kurata, Marina Hirose, Yasutaka Ojima, Keinoshin Wada, Yasuyuki Shibuya

**Affiliations:** 1 DDS, PhD Assistant Professor, Department of Oral Maxillofacial Surgery, Nagoya City University, Graduate School of Medical Sciences, Nagoya, Japan; 2 DDS, Resident, Department of Oral Maxillofacial Surgery, Nagoya City University, Graduate School of Medical Sciences, Nagoya, Japan; 3 DDS, PhD, Research Fellow, Department of Oral Maxillofacial Surgery, Nagoya City University, Graduate School of Medical Sciences, Nagoya, Japan; 4 DDS, PhD, Professor, Department of Oral Maxillofacial Surgery, Nagoya City University, Graduate School of Medical Sciences, Nagoya, Japan

## Abstract

**Background:**

Several sinus floor elevation procedures for implant placement have been introduced. The present study aimed to evaluate the implants placed with Platelet-rich fibrin (PRF) alone in atrophic posterior maxillae and survival rates and the potential factors associated with implant loss.

**Material and Methods:**

This retrospective study evaluated 71 implants in 34 patients after 1-7 years’ follow-up time. Statistical models were used to determine the implant survival and the potential factors associated with loss.

**Results:**

Overall, 7 implants were lost, and the cumulative survival rate at 7 years by implant-based and patient-bases analyses were 85.5% and 85.7%, respectively. The mean residual bone height (RBH) was 4.26 mm. The implant survival rate was significantly lower at RBH < 4 mm than RBH ≥ 4 mm.

**Conclusions:**

This retrospective study showed that sinus floor elevation with PRF alone could be applied in cases of lower RBH. However, it should be carefully performed in cases of RBH < 4 mm before surgery.

** Key words:**Platelet-rich fibrin, dental implant, sinus augmentation, retrospective study.

## Introduction

In the posterior maxillary area, several sinus floor elevation procedures for implant placement have been introduced since the 1980s. With the development of sinus elevation techniques, various graft materials for sinus augmentation have been used as filler to maintain adequate space for new bone formation. Although autogenous bone is still considered the gold standard, its use necessarily creates another wound at the donor site, such as the mandibular ramus or symphysis. Autogenous bone is therefore not widely used in clinical practical. Other graft materials, such as allogenic, xenogenic, alloplastic materials or combinations of different graft materials, have been introduced (1). However, these materials also have their limitations, including risk of infection, insufficient bone regeneration and increased overall cost. As such, no graft material appears to be superior to others at present.

Autologous platelet concentrates have been used for over 30 years and have the potential to induce wound healing (2). Whitmann *et al.* first reported the application of platelet-rich plasma (PRP) for oral surgery procedures (3). Although several studies of PRP have been reported, PRP application is complicated and associated with risk because of the use of bovine thrombin. In contrast, the application of platelet-rich fibrin (PRF), developed in 2001 by Choukroun *et al.*. as a second-generation platelet concentrate, is relatively simple, cheap and safe (4). Therefore, PRF has been applied for maxillary sinus augmentation. Previous reports have shown that sinus floor elevation with PRF as graft material achieved favorable results. Sinus augmentation using freeze-dried bone allograft mixed with PRF showed accelerated bone regeneration and reduced healing time (5). PRF and bovine bone graft material combination is also more effective in the first phases of wound healing than bovine bone graft material and collagen membrane combination (6). Recently, the use of PRF as a sole graft material for sinus augmentations with simultaneous implant placement has shown promising results. In 2008, Diss *et al.* first published the one-year results of implant placement with the osteotome technique using PRF as the only graft material (7). Toffler *et al.* showed that osteotome sinus floor elevation with PRF was extremely safe and successful at sites 5- to 8 mm residual bone height (RBH) (8). However, the longer-term outcomes of the procedure and failure factors have not been sufficiently examined. The aim of this retrospective study was to test the null hypothesis that there is no difference in failure factors for implants inserted with PRF as the only grafting material for sinus augmentation.

## Material and Methods

-Study design and patient selection

A retrospective study design was employed. The study protocol was approved by the Ethics Committee (No. 46-10-0004). The clinical procedures were performed in accordance with the Helsinki Declaration (revised in 2008), and all patients signed the informed consent forms before treatment.

The patients treated sinus floor augmentation using PRF as the sole grafting material at , from September 2010 to October 2015 were screened in this study. The patients’ medical and dental histories were checked at the initial appointment, and patients were then selected based on following criteria: 1) tooth loss in the maxillary posterior region, 2) good general health or controlled medical conditions, 3) implant placement by sinus floor elevation with PRF alone as the grafting material, 4) informed consent provided and 5) follow-up visit performed after implant placement at our hospital.

-Surgical and prosthetic procedure

Panoramic radiography and computer tomography (CT) were performed to examine the bone and sinus conditions. Surgery was performance under local anesthesia. A midcrestal incision was made, and the buccal and palatal mucoperiosteal flaps were reflected using a full-thickness approach to expose the posterior maxillary edentulous area. For the crestal approach, implant holes were drilled using the CAS-KIT (OSSTEM Implant Co., Busan, Korea). In accordance with the manufacturer’s guideline, the CAS-drill must be used with a stopper. Once the drill tip reached the Schneiderian membrane, saline was slowly injected into the prepared hole with pumping to detach the Schneiderian membrane by hydraulic pressure using a hydraulic membrane lifter (OSSTEM Implant Co., Busan, Korea). PRF clots were compressed into a thin membrane in accordance with the protocol of Choukroun * et al.* (5) and multiple PRF membranes were inserted into the sinus floor elevated site. Implants were inserted simultaneously. For the lateral approach, full-thickness flaps were elevated after a midcrestal incision was made. A lateral window via the buccal maxillary wall was created using the LAS-KIT (OSSTEM Implant Co., Busan, Korea). After careful elevation of the Schneiderian membrane, the bone window served as a new sinus floor. Two or three PRF clots were inserted into the sinus cavity. The implant sites were prepared with careful drilling, and then the implants were inserted. Finally, the PRF membrane was used to cover bony window site.

Furthermore, when performing a lateral approach to sinus floor elevation for delayed implant placement, a full-thickness mucoperiosteal flap was elevated, and then the lateral maxillary wall was carefully removed and the Schneiderian membrane was elevated with sinus curettes. After the intact sinus cavity was created, three or four PRF clots were inserted into the new sinus cavity. PRF membrane was used to cover the bony window site, and the flaps were sutured. After an approximately three-month healing period, implants were placed using the crestal approach. Three different sinus floor elevation procedures which are simultaneously carried out with the crestal and lateral approach, and staged implant placement were classified in this study. After the healing period, implant-supported supra-structures, including single crowns, fixed partial denture and overdentures, were fabricated and delivered to the patients. 

-Outcome measurements

• The implant survival

The implant survival criteria suggested by Buser * et al.* (9) were adopted, as follows: 1) the absence of implant mobility, 2) the absence of pain or any subjective sensation, 3) the absence of recurrent peri-implant infection and 4) the absence of continuous radiolucency around the implants. Implant failure was classified into two groups: early failure before loading, and late failure after loading (10). The implant survival rate was calculated by measuring the time elapsed from implant placement to the last follow-up visit or implant removal.

• Radiographic assessment

Radiographic assessments were conducted on CT images using a software program (Simplant, Dentsply, MA, USA) on a millimeter scale. Radiographs were analyzed by one examiner (MS). Before surgery, the RBH at the radiographic guides with a marker site was determined.

-Statistical analyses

The implant survival rate analyzed using the Kaplan-Meier curve. A logistic regression analysis was use to investigate the potential factors influencing implant loss. The independent factors were sex, age, smoking status, site (premolar or molar), and implant length (< 10 or ≥ 10 mm). The SPSS 17.0 software program (SPSS Inc., Chicago, IL, USA) was used to perform all statistical analyses, and the significance level was set to *p* < 0.05.

## Results

-Patient and implant information

Between September 2011 and October 2015, a total of 34 patients (17 male and 17 female), aged 29 to 82 years old (mean age 57.6), underwent sinus augmentation by PRF alone in this study. The average follow-up time was 3.43 years. The patients were treated with 71 implants inserted into 19 premolars and 52 molars. The implants were as follows: 21 POIEX implants, 3.7 to 5.2 mm in diameter and 8 to 10 mm in length (KYOCERA Medical, Osaka, Japan); 45 TSIII implants, 4.0 to 5.0 mm in diameter and 8.5 to 11.5 mm in length (OSSTEM Implant Co., Busan, Korea); 4 USIII implants, 4.0 to 5.0 mm in diameter and 8.5 to 11.5 mm in length (OSSTEM Implant Co., Busan, Korea); 1 GSIII implant, 4.5 mm in diameter and 10 mm in length (OSSTEM Implant Co., Busan, Korea).

Fifty-four implants were placed simultaneously via the crestal approach, and 15 were placed simultaneously via the lateral approach. Two implants in one patient were placed via the staged approach. The distributions of patient and implant are presented in  Table 1.

**Table 1:**
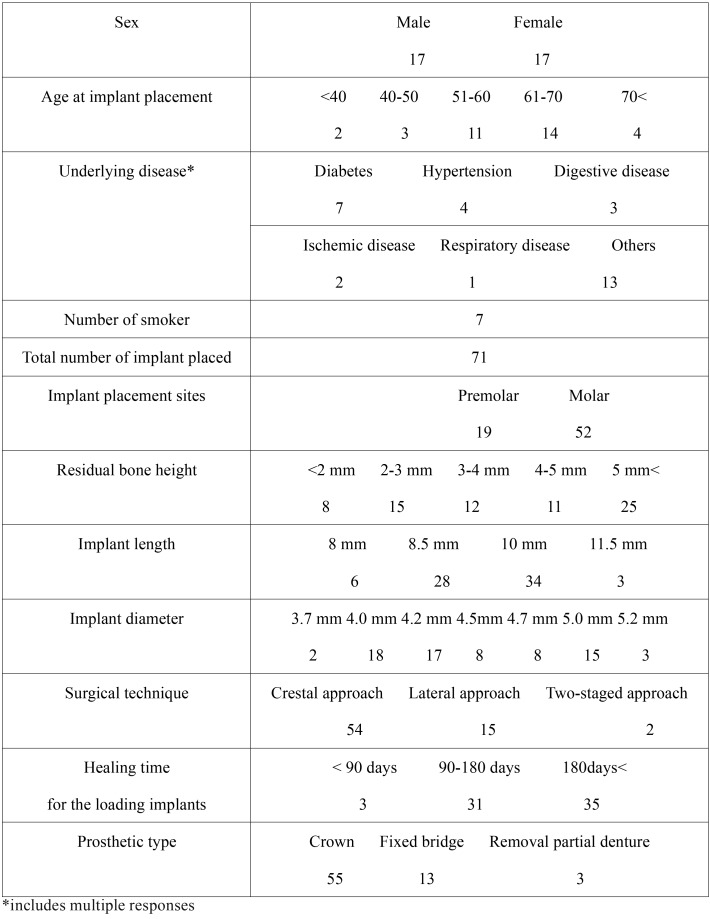
Distribution of patients and implants.

-The implant survival and failures

**Figure 1:**
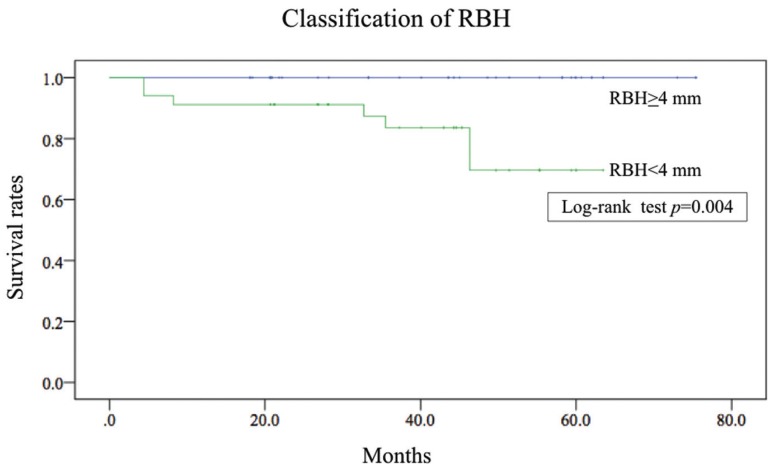
Kaplan-Meier survival curves for RBH ≥ 4 mm and RBH < 4 mm by an implant-based analysis. RBH: Residual bone height.

After 1-7 years follow-up, seven implants in four patients were lost. The cumulative survival rates were 85.5% by implant-based analysis and 85.7% by patient-based analysis  Table 2. Moreover, the cumulative survival rates were 100% and 69.6% for the RBH ≥ 4 mm group and the RBH < 4 mm group, respectively (Log-rank test: *p* = 0.004) Figure 1. The details regarding the lost implants are shown in  Table 3. Among the seven lost implants, four implants in two patients were lost two years after placement. One of them was removed and replaced with sinus augmentation by PRF alone. Another three implants in two patients were lost before prosthetic loading.

**Table 2:**
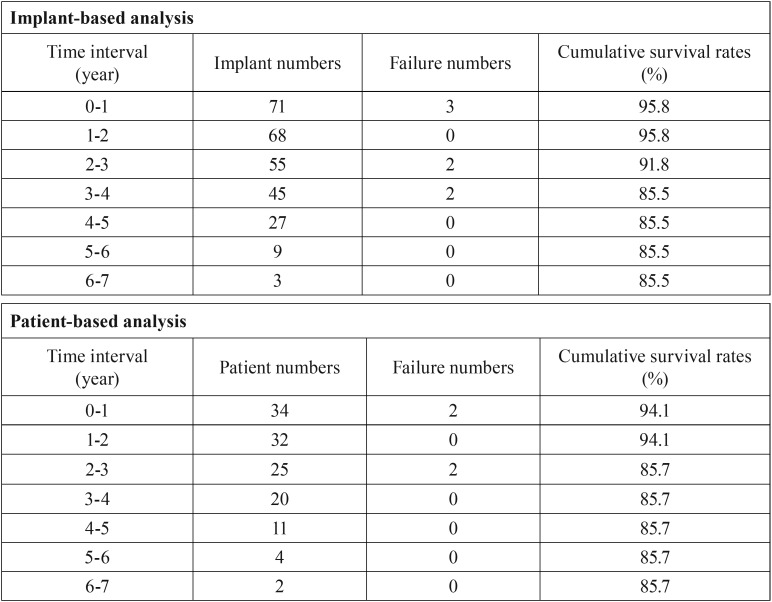
The cumulative survival rate of implant- and patient-based analysis.

**Table 3:**
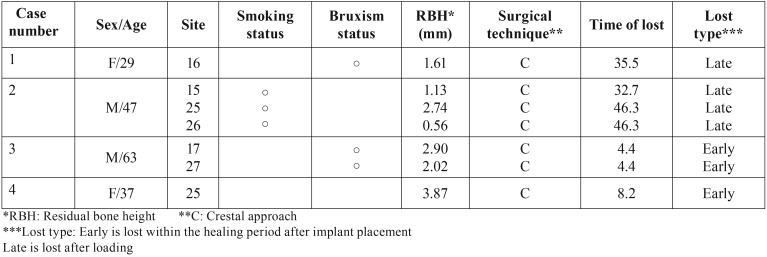
The details of the lost implant.

**Table 4:**
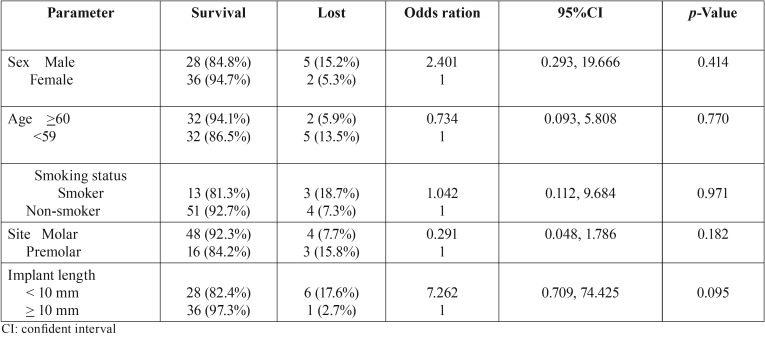
Logistic regression for determining the factors affecting implant loss after sinus augmentation with Platelet-rich fibrin alone.

-Radiographic assessments

The mean RBH before implant placement as measured in the radiographs was 4.26 ± 2.11 mm and ranged from 0.56 to 9.60 mm in this study.

-Logistic regression analysis of risk indicators for implant loss

Logistic regression for determining the factors affecting implant loss are showed in  Table 4. However, implant loss was found for male patients (OR, 2.4) and implant length< 10 mm (OR, 7.3), none of the factors was found be significantly related to implant loss.

## Discussion

Various sinus augmentation procedures have been introduced to overcome the insufficient bone height for implant placement in the posterior maxillae. The procedures with platelet concentrates have been developed. PRP has been widely used as bone graft material in sinus augmentation procedures. However, Lemos *et al.* found that PRP with bone graft appeared to have no influence on bone formation or the implant survival in maxillary sinus augmentation (11). Furthermore, preparation for PRP is relatively complex, and there are potential risks with this material, as PRP contains synthetic or anticoagulant materials. Therefore, the actual efficacy of PRP is controversial, although it has the ability to release various growth factors to enhance bone regeneration.

PRF has several advantages over PRP, including easier preparation and lower cost (6). In addition, although the *in vitro* conditions do not fully reflect the clinical situation, the biological characteristics of PRF are also superior to PRP (12). Accordingly, there have been some published clinical reports of sinus floor elevation using PRF as grafting material (5,7,8,13). Choukroun et al reported sinus floor augmentation using PRF in combination with a freeze-dried bone allograft (5). In that report, the mixed graft material was able to reduce the healing time. Tanaka *et al.* also described increased new bone formation in the histological evaluation of sinus augmentation with deproteinized bovine bone mixed with PRF (14). However, combination with these particulate bone materials has been associated with increased costs and risk of infection. Accordingly, sinus floor elevation using PRF as the sole graft material has been recently introduced. Diss *et al.* reported the survival rate at 1 year after sinus floor elevation with PRF as the sole graft material based on 20 patients and 35 implants (7). Subsequently, Toffler reported the survival rate of 138 implants in 110 patients at less than 12 months after placement using a similar procedure (8). These survival rates were 97.1% and 97.8%, respectively (7,8).

The present retrospective study evaluated the outcomes of 71 implants in 34 patients treated with sinus augmentation with PRF alone after 1-7 years’ follow-up. A 7-year cumulative survival rate of about 85.5% was detected. This result was slightly lower than the values described by Diss and Toffler (7,8). However, in those two studies, the follow-up duration from implant placement was shorter (one year or less) than in the present study. Furthermore, the mean RBH differed from the present study (6.5 and 6.6 mm, respectively, in Diss and Toffler’s studies, and 4.3 mm in the present study). For implants placed using sinus floor elevation, the survival rate is affected by the preexisting bone height between the sinus floor and crest (15). Therefore, the lower survival rate of the present study than these reports was affected by the RBH.

Grafting bone materials, such as a xenograft, help to maintain the membrane in an elevated position, since they have strong physical properties (16). While whether or not PRF can maintain the membrane in an elevated position is unclear, since they have softer physical properties. Therefore, the volume and the form of PRF placed into sinus cavity might make it difficult to maintain an elevated membrane position. The elevated membrane position sequentially drops down to the implant apex. The implant is similar in appearance to a tent pole. However, Kanayama *et al.* found that membranes elevated using PRF alone were able to maintain a higher position above the implant apex, a result which may have been influenced by many factors, including the shape of the sinus cavity, the presence or absence of adjacent teeth and other factors (17).

A logistic analysis was performed to investigated the factors associated with implant loss. There were no significant differences in the sex, age, smoking status, placement site or implant length between cases of failure and success. A previous study reported on the relationship between RBH and implant failure/success with the sinus floor elevation procedure (15,18,19). Del Fabbro also described a favorable prognosis when the RBH was at least 5 mm (18). A study by Toffler using a similar procedure with PRF also described a decreased survival rate when RBH was <5 mm (19). Rosen found that the survival rate decreased to 85.7% when RBH was ≤ 4 mm.15 These results suggest a potentially favorable prognosis with a line of demarcation between RBH of 4 or 5 mm. Therefore, we classified subjects into two groups (RBH ≥ 4 mm and < 4 mm) and investigated the survival rate. When RBH was ≥ 4 mm, the survival rate was 100%. In contrast, when RBH was < 4 mm, the survival rate dropped to 69.6%. Furthermore, the Therefore, RBH also affects the survival of the implant when sinus augmentation with PRF alone is applied. Implant loss in cases of lower RBH occurs due to difficulty in acquiring implant stability. In addition, three of the four cases of loss in the present study were found to have smoking and bruxism, factors that may be associated with an increased risk of implant loss (20,21).

Lundgren *et al.* first suggested the placement of implants without the addition of any grafting materials (22). Furthermore, four randomized clinical trials and a systematic review for a procedure without bone graft have also been reported and indicated favorable outcomes, such as solicitude regarding remaining graft particles and reduced rates of complications and lower costs (10,23-25). However, this graft-free procedure still remains controversial. Nedir showed that sites with RBH < 4 mm allowed implant placement without the use of grafting materials, although a greater amount of new bone formation was gained when grafting materials were used (26). However, in the present study, all cases of failure showed RBH < 4 mm. Therefore, to evaluate how many millimeters of RBH are required without grafting materials, larger samples and longer-term observations are still needed.

Regarding the advantage of using PRF over procedures without grafting materials, PRF could enhance bone generation (27). Perforation of the Schneiderian membrane is the most common complication in sinus grafting procedures, occurring in 10% to 60% of cases (28,29). We believe that PRF presents a great advantage with respect to protecting the membrane from perforation when placing implants, acting as a kind of “barrier material”. During implant placement without graft materials, there is some possibility of the implant apex perforating the membrane after elevating sinus floor. Since the crestal approach for sinus floor elevation is a blind procedure and it is difficult to confirm whether or not perforation has occurred, the use of any graft materials might not be favorable. PRF also affects membrane repair if sinus membrane perforations occur during the sinus lift procedure (7,30). The present study included a two-stage approach with PRF alone. PRF as a grafting material is responsible for maintaining the Schneiderian membrane position. PRF is absorbed gradually, so PRF as the sole filling material without simultaneous implant placement may not be adequate for maintaining the membrane. However, our previous report disputes this notion (13). That report showed that PRF clots alone can create space between the original sinus floor and the elevated Schneiderian membrane. The volume of PRF may be important for maintaining the membrane. Therefore, PRF clots may be more useful than a PRF membrane. However, the two-stage protocol has several limitations, like a relatively short follow-up period. Further studies are therefore needed to obtain stronger evidence to confirm our findings.

## Conclusion

This 1- to 7-year retrospective study showed that sinus floor elevation with PRF alone is a safe procedure because PRF was able to protect the membrane during implant installation and thereby avoid any perforation of the membrane. The procedure provides favorable results when the RBH is low. However, the risk of implant loss increases when RBH is < 4 mm. These findings suggest that long-term follow-up and a large sample are required to confirm the predictability of this procedure.

## References

[1] Sohn D, Moon JW,  Moon KN,  Cho SC,  Kang PS (2010). New bone Formation in the maxillary sinus using only absorbable gelatin sponge. J Oral and Maxillofac Surg.

[2] Borie E, Oliví DG,  Orsi IA,  Garlet K,  Weber B,  Beltrán V (2015). Platelet-rich fibrin application in dentistry: a literature review. Int J Clin and Exp Med.

[3] Whitmann DH, Berry RL, Green DM (1997). Platelet gel: an alternative to fibrin glue with applications in oral and maxillofacial surgery. J Oral Maxillofac Surg.

[4] Choukroun J, Adda F, Schoeffler C, Vervelle A (2001). An opportunity in perio-implantology: The PRF (in French). Implantodontie.

[5] Choukroun J, Diss A,  Simonpieri A,  Girard MO,  Schoeffler C,  Dohan SL (2006). Platelet-rich fibrin (PRF): A second- generation platelet concentrate. Part V: Histologic evaluations of PRF effects on bone allograft maturation in sinus lift. Oral Surg Oral Med Oral Pathol Oral Radiol Endod.

[6] Bolukbasi N, Ersanlı S,  Keklikoglu N,  Basegmez C,  Ozdemir T (2015). Sinus augmentation with platelet-rich fibrin in combination with bovine bone graft versus bovine bone graft in combination with collagen membrane. J Oral Implantol.

[7] Diss A, Dohan DM,  Mouhyi J,  Mahler P (2008). Osteotome sinus floor elevation using Choukroun's platelet-rich fibrin as grafting material: a 1-year prospective pilot study with microthreaded implants. Oral Surg Oral Med Oral Pathol Oral Radiol Endod.

[8] Toffler M, Toscano N, Holtzclaw D (2010). Osteotome-mediated sinus floor elevation using only platelet-rich fibrin: An early report on 110 patients. Implant Dent.

[9] Buser D, Mericske-Stern R,  Bernard JP,  Behneke A,  Behneke N,  Hirt HP (1997). Long-term evaluation of non-submerged ITI implants. Part 1: 8-year life table analysis of a prospective multi-center study with 2359 implants. Clin Oral Implants Res.

[10] Si MS, Shou YW,  Shi YT,  Yang GL,  Wang HM,  He FM (2016). Long-term outcomes of osteotome sinus floor elevation without bone grafts: a clinical retrospective study of 4-9 years. Clin Oral Implants Res.

[11] Lemos CA, Mello CC,  dos Santos DM,  Verri FR,  Goiato MC,  Pellizzer EP (2016). Effects of platelet-rich plasma in association with bone grafts in maxillary sinus augmentation: a systematic review and meta-analysis. Int J Oral Maxillofac Surg.

[12] He L, Lin Y,  Hu X,  Zhang Y,  Wu H (2009). A comparative study of platelet-rich fibrin(PRF) and platelet-rich plasma(PRP) on the effect of proliferation and differentiation of rat osteoblasts in vitro. Oral Surg Oral Med Oral Pathol Oral Radiol Endod.

[13] Aoki N, Kanayama T,  Maeda M,  Horii K,  Miyamoto H,  Wada K (2016). Sinus augmentation by platelet-rich fibrin alone: A report of two cases with histological examinations. Case Rep Dent.

[14] Tanaka H, Toyoshima T,  Atsuta I,  Ayukawa Y,  Sasaki M,  Matsushita Y (2015). Additional effects of platelet-rich fibrin on bone regeneration in sinus augmentation with deproteinized bovine bone mineral: Preliminary results. Implant Dent.

[15] Rosen PS, Summers R,  Mellado JR,  Salkin LM,  Shanaman RH,  Marks MH (1999). The bone-added osteotome sinus floor elevation technique: Multicenter retrospective report of consecutively treated patients. Int J Oral Maxillofac Implants.

[16] Kanayama T, Horii K, Senga Y, Shibuya Y (2016). Crestal approach to sinus floor elevation for atrophic maxilla using platelet-rich fibrin as only grafting material: A 1-year prospective study. Implant Dent.

[17] Kanayama T, Shibuya Y, Wada K (2016). Noticeable bone regeneration beyond the implant tip after a crestal approach sinus lift using only platelet rich fibrin: A report of two cases. OHDM.

[18] Del Fabbro M, Corbella S,  Weinstein T,  Ceresoli V,  Taschieri S (2012). Implant survival rates after osteotome-mediated maxillary sinus augmentation: A systematic review. Clin Implant Dent Relat Res.

[19] Toffler M (2004). Osteotome-mediated sinus floor elevation: a clinical report. Int J Oral Maxillofac Implants.

[20] Chrcanovic BR, Albrektsson T, Wennerberg A (2015). Smoking and dental implants: a systematic review and meta-analysis. J Dent.

[21] Chrcanovic BR, Kisch J,  Albrektsson T,  Wennerberg A (2016). Bruxism and dental implant failures: a multilevel mixed effects parametric survival analysis approach. J Oral Rehabil.

[22] Lundgren S, Andersson S, Sennerby L (2003). Spontaneous bone formation in the maxillary sinus after removal of a cyst: coincidence or consequence?. Clin Implant Dent Relat Res.

[23] Borges FL, Dias RO,  Piattelli A,  Onuma T,  Gouveia Cardoso LA,  Salomão M (2011). Simultaneous sinus membrane elevation and dental implant placement without bone graft: a 6-month follow-up study. J Periodontol.

[24] Nedir R, Nurdin N,  Khoury P,  Perneger T,  Hage ME,  Bernard JP (2013). Osteotome sinus floor elevation with and without grafting material in the severely atrophic maxilla. A 1-year prospective randomized controlled study. Clin Oral Implants Res.

[25] Nedir R, Nurdin N,  Khoury P,  Bischof M (2016). Short implants placed with or without grafting in atrophic sinuses: the 3-year results of a prospective randomized controlled study. Clin Implant Dent Relat Res.

[26] Nedir R, Nurdin N,  Abi Najm S,  El Hage M,  Bischof M (2016). Short implants placed with or without grafting in atrophic sinuses: the 5-year results of a prospective randomized controlled study. Clin Oral Implants Res.

[27] Ali S, Bakry SA, Abd-Elhakam H (2015). Platelet-rich fibrin in maxillary sinus augmentation: A systematic review. J Oral Implantol.

[28] Proussaefs P, Lozada J, Kim J,  Rohrer MD (2004). Repair of the perforated sinus membrane with a resorbable collagen membrane: a human study. Int J Oral Maxillofac Implants.

[29] Ardekian L, Oved-Peleg E,  Mactei EE,  Peled M (2006). The clinical significance of sinus membrane perforation during augmentation of the maxillary sinus. J Oral Maxillofac Surg.

[30] Choi BH, Zhu SJ,  Jung JH,  Lee SH,  Huh JY (2006). The use of autologous fibrin glue for closing sinus membrane perforations during sinus lifts. Oral Surg Oral Med Oral Pathol Oral Radiol Endod.

